# The value of computed tomography perfusion & transcranial Doppler in early diagnosis of cerebral vasospasm in aneurysmal & traumatic subarachnoid hemorrhage

**DOI:** 10.4155/fsoa-2018-0015

**Published:** 2018-06-26

**Authors:** George Fotakopoulos, Demosthenes Makris, Polikceni Kotlia, Effie Kapsalaki, John Papanikolaou, Iordanis Georgiadis, Epaminondas Zakynthinos, Kostas Fountas

**Affiliations:** 1 Department of Neurosurgery, University Hospital of Thessaly, University Hospital of Larissa, Biopolis, 41110 Larissa, Thessaly, Greece; 2 Department of Head of Critical Care, University of Thessaly, University Hospital of Larissa, Biopolis, 41110 Larissa, Thessaly, Greece; 3 Departments of Neurosurgery & Diagnostic Radiology, University Hospital of Larisa, Faculty of Medicine, University of Thessaly, Larissa, Greece

**Keywords:** cerebral vasospasm, CT perfusion, subarachnoid hemorrhage, transcranial Doppler

## Abstract

Early detection and diagnosis of cerebral vasospasm in subarachnoid hemorrhage may be challenging both on clinical and radiographic grounds. In this respect we conducted a pilot study in order to assess the feasibility of the technique in the everyday setting of a tertiary hospital and to evaluate the diagnostic performance of different diagnostic computed tomography perfusion aspects in diagnosing the clinical outcome of patients with subarachnoid hemorrhage. Receiver-operating characteristic analysis showed that a cerebral blood flow value of <24.5 presented 67% sensitivity and 100% specificity to diagnose adverse ischemic events at 1 month (p = 0.041). These case series data provide evidence that computed tomography perfusion-derived cerebral blood flow is a measurable index that may detect the degree of cerebral ischemia in a very early stage.

## Lay abstract

Early detection and diagnosis of cerebral vasospasm in subarachnoid hemorrhage is important but may be challenging both on clinical or radiographic grounds. This article provides evidence that computed tomography perfusion-derived cerebral blood flow is a measurable index that could detect the degree of cerebral ischemia in a very early stage in patients suffering with subarachnoid hemorrhage. Larger studies are needed in order to better define the role of computed tomography perfusion in early diagnosis of cerebral vasospasm.

Symptomatic cerebral vasospasm (CV) is the most important postoperative risk factor for poor outcome after subarachnoid hemorrhage (SAH) and, unfortunately, 16–65% of those patients develop ischemia [1–3], despite the use of any therapeutic strategy. A multiplicity of procedures is undertaken to improve cerebral blood flow (CBF) in SAH patients developing CV. These include hyperdynamic therapy, intra-arterial and intrathecal drug infusion, intra-aortic balloon counterpulsation, and new experimental methods; their combination may be very helpful if treatment is started in time [4]. If CV is missed or cure started too late, cerebral infarction might build up in spite of maximum treatment.

Early detection and diagnosis of CV in SAH may be challenging both on clinical or radiographic grounds [2]. Despite that 40–70% of patients have evidence of arterial narrowing (in angiography or Doppler ultrasound), only 20–30% develop the clinical syndrome [2]. Computer tomography provides useful information but it is limited to identifying the extent of irreversibly damaged tissue (ischemic core) [5–7], and to demonstrating that reperfusion therapy may be insignificant or even potentially harmful when the ischemic core is large, or the perfusion lesion is particularly severe [8,9]. On the other hand, computed tomography perfusion (CTP) can show abnormalities not detectable by other techniques and may be advantageous for the evaluation of cerebral ischemia (CI) associated with SAH [10]. However, it is questionable whether CPT performance is feasible in the everyday practice, or if it can be used as a stand-alone diagnostic tool without additional MRI data for clinical outcome prognosis in SAH patients [11,12], in addition, data regarding its relationship with other noninvasive methods such as transcranial Doppler (TCD) are limited [13,14].

In this respect we conducted a pilot study in order to assess the feasibility of the technique in the everyday setting of a tertiary hospital and to evaluate the diagnostic performance of different diagnostic CTP aspects in diagnosing the clinical outcome of patients with SAH (traumatic and aneurysmal).

## Case presentation

### Material & methods

This was a prospective case series study. Patients with spontaneous SAH or traumatic SAH who were admitted between July and September 2016 were included in the study if they presented Glasgow Coma Scale of 15/15 to 4/15; computed tomography (CT) showing high-grade SAH in terms of neuroimaging severity (Fisher grading IV) within the first 48 h from SAH. Patients with risk factors for CI other than vasospasm, that is, patients with arrhythmias or atrial fibrillation, hypotension, diabetes, hyperlipidemia or, patients with risk factors for hemorrhage, that is, therapy with anticoagulants, serious hepatic disease, were excluded from the study. Informed consent was obtained from all participants or the next of kin.

Participants were followed until discharge or for 30 days if discharge occurred earlier. Clinical outcomes were recorded at 30 days by using CT scan (performed by two radiologists) and full neurological examination including Glasgow Coma Scale assessment (performed by two physicians other than the treating physicians). Clinical outcome was classified as normal, when no neurological sequelae +/or radiologic evidence of ischemic abnormalities were detected or, adversely, when neurologic or radiologic abnormalities were diagnosed +/or, death.

CTP was performed on the third to sixth day, in order to detect a measurable index for CI after vasospasm since angiographically visible cerebral arteries narrowing (vasospasm) after SAH is most frequently present 3/4–10 days after SAH. CBF and cerebral blood volume (CBV) values were recorded and evaluated using two adjacent 10 mm slices positioned at the level of the basal ganglia with the same angulation as for native CT. A bolus of 50 ml of nonionic contrast medium (Imeron 400, Bracco, Konstanz, Germany) was administered by a power injector into a central venous catheter at a flow rate of 4 ml/s followed by 30 ml of saline. Four seconds after beginning of the bolus, 40 images were collected at each slice level at a rate of two images per second (120 kV, 110 mAs, matrix 512 × 512). CTP parameters were calculated by commercially available postprocessing software platform (Perfusion CT, Siemens, Munich, Germany) and CTP color maps were qualitatively assessed using a visual grading scale. A positive visual assessment was noted for side-to-side asymmetries or clear bilateral defects suggesting a decrease in CBF, CBV, mean transit time (MTT), which were related by the central volume principle: CBF = CBV/MTT [15]. CBV was measured in units of milliliters of blood per 100 g of brain and was defined as the volume of flowing blood for a given volume of brain [16]. MTT was measured in seconds and defined as the average amount of time it takes blood to transit through the given volume of brain.

On the same day, 15–20 min before CPT, a TCD via transtemporal window was performed to visualize and evaluate flow velocities in middle, anterior, posterior and posterior communicant arteries (Middle cerebral artery [MCA], anterior cerebral artery [ACA], posterior cerebral artery [PCA] and posterior communicate artery [PCOM]). Peak systolic velocity (PSV), defined as the maximum value (in cm/s) of flow velocity in systole, was calculated at the apex of the waveform. End-diastolic velocity (EDV in cm/s) was measured at end diastole, usually at the lowest point before the onset of a new waveform begins which lies between 20 and 50% of the PSV values [17]. The Lindegaard ratio, defined as MCA mean CBF velocity divided by extracranial internal carotid artery mean cerebral flow velocity, was used as an index less affected by systemic hemodynamic variations. Mean flow velocity (MFV in cm/s) was estimated as the average of the edge frequency over a cardiac cycle, which was calculated as EDV plus a third of the difference between PSV and EDV (MFV [cm/s] = [PSV+2EDV]/3) [17]. Sonographic vasospasm was defined as an MFV >140 cm/s in the MCA, ACA and/or PCA or a MFV of >90 cm/s in the basilar artery. Pulsatility Index (PI) = PSV - EDV/MFV was used as an index for elevated intracranial pressure [17].

### Statistical analysis

Data are presented as mean standard error. Receiver operating characteristic (ROC) analysis was performed to demonstrate the performance of CTP or TCD indices in diagnosing adverse outcome. A p < 0.005 value was considered as significant. Statistical Package for the Social Sciences (SPSS.11, IL, USA) was used for analysis.

### Results

Seven patients were included in this case series study [Table 1]. Two of them had traumatic SAH, three had aneurysmal SAH and two patients who presented no SAH were used as controls. Baseline characteristics and outcomes of study participants are shown in Table 1. Overall, three subjects (subjects 1, 3 and 4) presented neurologic sequelae at 1 month.

**Table 1.** **Baseline characteristics of patients and clinical outcome.**

**Parameters**	**S1 (tSAH)**	**S2 (tSAH)**	**S3 (aSAH)**	**S4 (aSAH)**	**S5 (aSAH)**	**S6 (without SAH)**	**S7 (without SAH)**
Age	68	16	68	41	55	69	85

Sex	M	M	F	M	F	M	F

GCS	8	13	13	12	14	7	13

Fisher scale	2	2	4	3	3	1	1

Cause of admission	TBI aEDH	TBI	PCOM aneurysm	ACOM aneurysm	Left MCA aneurysm	TBI without SAH	TBI without SAH

Clinical outcome	Right leg palsy	–	–	Emotional disturbances	–	–	–

Radiologic finding (CT scan)	Ischemic lesion to the left parietal lobe	–	Ischemic lesion to the right temporoparietal	Small area with ischemic lesion to the right frontal	–	–	–

ACOM: Anterior communicate artery; aEDH: Acute epidural hematoma; aSAH: Aneurismal subarachnoid hemorrhage; CT: Computer tomography; F: Female; GCS: Glashow Coma Scale; M: Male; MCA: Middle cerebral artery; PCOM: Posterior communicate artery; SAH: Subarachnoid hemorrhage; tSAH: Traumatic subarachnoid hemorrhage; TBI: Traumatic brain injury.

### Transcranial Doppler

**Figure 1.**
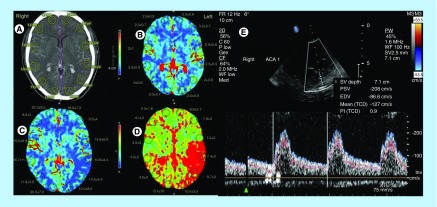
**A 41-year-old male patient (S4 patient in text) with subarachnoid hemorrhage caused by rupture of anterior communicating artery aneurysm, developed vasospasm and 8 days after the bleeding had small area with ischemic lesion to the right frontal.** **(A)** Computed tomography scan in the second day after rupture, showed aneurismal subarachnoid hemorrhage without ischemia, computed tomography perfusion in the same time. **(B)** Blood volume map; **(C)** cerebral blood flow map and **(D)** time to peak map images in the same patient showed normal brain parenchyma and regional cerebral blood volume, but abnormal regional cerebral blood flow and mean transit time, suggesting an area of low perfusion; **(E)** transcranial Doppler in the right anterior cerebral artery; pulsatility index was normal (PI = 0.9).

TCD data are presented in Table 2. In subject 1, TCD revealed increased PI in the right PCA right (1.7) and right ACA (1.9) and normal PI at MCA. The PI in the left ACA was also increased (1.7). In subject 3, TCD showed elevated PSVs and PIs into the left PCA, MCA and ICA (134, 118, 120 m/s and 1.5, 1.4, 1.7, respectively) and increased PSV = 190 m/s with normal PI = 0.9 in the right ACA. In subject 4, TCD presented increased PSV and PI in the left PCA and MCA (118 and 199 m/s and 1.5 and 1.1, respectively), but in the right ACA PI was normal (PI = 0.9) (Figure 1).

**Table 2.** **Transcranial Doppler parameters.**

	**S1 (tSAH)**	**S2 (tSAH)**	**S3 (aSAH)**	**S4 (aSAH)**	**S5 (aSAH)**	**S6 (without SAH)**	**S7 (without SAH)**
PCA (left)							

PSV (cm/s)	81.2	63.4	134	118	121	57.1	56.2

EDV (cm/s)	34.2	30.2	32.1	28.8	30.2	17.4	15

MFV (cm/s)	49.8	42.2	66	58.5	53.8	28.4	28.7

PI	0.9	0.7	1.5	1.5	1.6	1.3	1.4

PCA (Right)							

PSV (cm/s)	98.3	68.5	87.4	94	86.7	45.8	42.3

EDV (cm/s)	19.3	36.8	35.1	30.4	40.2	15	16

MFV (cm/s)	46	47	49.3	52	54.2	25	26

PI	1.7	0.7	1.1	1.2	0.8	1.2	1

MCA (Left)							

PSV (cm/s)	91.1	106	118	199	168.2	59.9	64

EDV (cm/s)	34.2	51.2	31	74.5	46.7	18.3	19

MFV (cm/s)	53.1	69	60	116	87.2	32	35.2

PI	1.0	0.8	1.4	1.1	1.3	1.3	1.28

MCA (Right)							

PSV (cm/s)	71.1	82.8	113	188	190	81.8	77.3

EDV (cm/s)	32.1	47.5	33.2	64.3	49.8	24.2	27

MFV (cm/s)	45.1	59	60	106	96.8	43	43.7

PI	0.8	0.6	1.3	1.1	1.4	1.3	1.1

ICA (left)							

PSV (cm/s)	85.5	77.1	89.3	66.3	75.3	57.7	52.1

EDV (cm/s)	23	24.2	29	23.5	37.8	13.8	16

MFV (cm/s)	43.8	41.8	49.1	37.8	50.3	28.4	20

PI	1.4	1.3	1.3	1.1	0.75	1.55	1.8

Left Lindegaard Index (mMCA/mICA)	2.5	1.65	1.22	3.06	1.77	1.12	1.76

ICA (right)							

PSV (cm/s)	179	116	120.3	48.3	109.3	75.3	63.2

EDV (cm/s)	36.4	37.3	24.1	19.5	29.8	17.2	17.8

MFV (cm/s)	84	63.5	56.2	29.1	56.3	33.6	32.9

Right Lindegaard Index (mMCA/mICA)		0.92	1.06	3.64	1.68	1.28	1.16

PI	1.7	1.24	1.7	1.0	1.4	1.7	1.4

ACA (left)							

PSV (cm/s)	138	88	94.1	143	126	83.5	79.1

EDV (cm/s)	28.4	40.7	26.1	54.4	48.9	22.7	24

MFV (cm/s)	64.9	56.4	48.7	84	74.6	43	42.3

PI	1.7	0.8	1.4	1.0	1	1.4	1.3

ACA (right)							

PSV (cm/s)	468	145	190	208	165.4	76	82

EDV (cm/s)	77.9	73.8	79.2	86.8	89.2	27.6	29.1

MFV (cm/s)	208	98	119	127	114	44	46.7

PI	1.9	0.7	0.9	0.9	0.6	1.1	1.1

ACA: Anterior cerebral artery; aSAH: Aneurismal subarachnoid hemorrhage; EDV: End-diastolic velocity; ICA: Internal carotid artery; MCA: Middle cerebral artery; MFV: Mean flow velocity; mICA: Mean velocity in the Internal Carotid Artery; mMCA: Mean velocity in the Middle Cerebral Artery; PCA: Posterior cerebral artery; PI: Pulsatility index; PSV: Peak systolic velocity; TCD: Transcranial Doppler; tSAH: Traumatic subarachnoid hemorrhage.

### Computed tomography perfusion

CTP data are presented in Table 3. In Subject 1, CTP showed reduced CBF (in left frontal and parietal, right frontal areas), normal CBV values and prolonged MTT (7.4 ± 2 to 10.5 ± 3 s), possibly due to the compression of the acute epidural hematoma in the right hemisphere. In subject 3, CTP revealed reduced CBV and CBF in right frontal, parietal areas and prolonged MTT mainly in the left side (7.4 ± 2 to 10.9 ± 3 s). In subject 4, CTP showed reduction of CBF and prolonged MTT on the left side from (7.4 ± 2 to 8.7 ± 2 s) (Figure 1).

**Table 3.** **Computed tomography perfusion (white matter) parameters.**

**CBV (ml/100 g)**	**S1 (tSAH)**	**S2 (tSAH)**	**S3 (aSAH)**	**S4 (aSAH)**	**S5 (aSAH)**	**S6 (without SAH)**	**S7 (without SAH)**
Right							

Frontal	2.3 ± 0.7	4.3 ± 2	1.4 ± 0.5	2.3 ± 0.7	3.1 ± 0.1	3.8 ± 0.7	4.1 ± 0.3

Parietal	2.5 ± 1	4.2 ± 2	2.2 ± 0.6	2 ± 1	4.4 ± 0.4	3.6 ± 0.9	3.7 ± 0.2

Occipital	2 ± 0.8	5 ± 2.3	3.5 ± 0.5	2 ± 0.8	3.9 ± 0.2	4 ± 0.8	3 ± 0.6

Left							

Frontal	3.2 ± 0.7	4.4 ± 2.5	2.5 ± 0.9	3.2 ± 0.7	2.2 ± 0.5	4.8 ± 0.7	2.4 ± 2.3

Parietal	3.9 ± 0.8	4.3 ± 1.8	3.1 ± 0.7	2.9 ± 0.8	4.1 ± 0.6	4.5 ± 0.7	3.5 ± 1.8

Occipital	2 ± 0.7	5.2 ± 2.3	2.3 ± 0.4	2 ± 0.7	2.8 ± 0.3	3.9 ± 0.9	3.1 ± 2.4

CBF (ml/100 g/min)							

Right							

Frontal	18.6 ± 4	32.8 ± 6	4.2 ± 2.3	8.6 ± 4	34.1 ± 1.4	42.8 ± 4.5	53 ± 5.2

Parietal	35.6 ± 8.8	30 ± 13	10.6 ± 3.9	15.6 ± 8.8	33.3 ± 1.5	12 ± 7	41 ± 11

Occipital	31.8 ± 5.9	30 ± 13	17.4 ± 5.6	11.8 ± 3.9	33.4 ± 0.7	31.9 ± 3.3	48 ± 10

Left							

Frontal	19.5 ± 4.2	33.2 ± 5	5.3 ± 2.8	9.5 ± 4	16.3 ± 1.7	44.7 ± 5.5	42.3 ± 4.7

Parietal	15.9 ± 6.9	33 ± 13	12.9 ± 4	15.9 ± 6.9	11.4 ± 0.9	42.9 ± 7	41 ± 12

Occipital	34.5 ± 5.5	33 ± 15	17.5 ± 4.1	14.5 ± 5.5	36.1 ± 2	41.4 ± 5	35 ± 14

MTT (s)							

Right							

Frontal	9.1 ± 3.5	7.3 ± 4	5 ± 2.5	4.1 ± 3	10.9 ± 1.1	4 ± 2.5	3.9 ± 0.4

Parietal	8.2 ± 3.2	9.3 ± 2.1	7.4 ± 3.3	4.2 ± 3	8.7 ± 2.1	3.9 ± 3	4 ± 2.1

Occipital	10.5 ± 3	10 ± 2.8	6 ± 1.5	4.5 ± 3	7.1 ± 1.1	4 ± 2.6	4.9 ± 2.7

Left							

Frontal	7.9 ± 3.3	7.4 ± 3	5.3 ± 4.1	7.9 ± 3	9.1 ± 1.9	3.9 ± 3.3	3.8 ± 2.9

Parietal	7.4 ± 2	9.6 ± 2	2.5 ± 0.9	7.4 ± 2	6 ± 0.9	4.7 ± 2.6	4.1 ± 2

Occipital	8.7 ± 2	9.7 ± 2	6.3 ± 1.3	8.7 ± 2	5.2 ± 1.1	3.8 ± 3	4.2 ± 3

aSAH: Aneurismal subarachnoid hemorrhage; CBF: Cerebral blood flow; CBV: Cerebral blood volume; CTP: Computer tomography perfusion; MTT: Mean transit time; SAH: Subarachnoid hemorrhage; tSAH: Traumatic subarachnoid hemorrhage.

### Diagnostics

**Figure 2.**
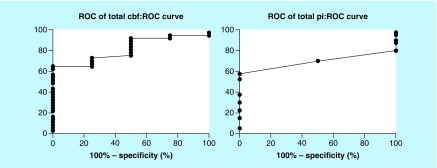
**Receiver-operating characteristic curve.** Receiver-operating characteristic analysis showed that cerebral blood flow presented the best performance among computed tomography perfusion and transcranial Doppler variables (Pulsatility Index) assessed with an area under curve standard error AUC(SE) of 0.81(0.08), p = 0.041 (Figure 2); a cerebral blood flow value of <24.5 presented 67% sensitivity and 100% specificity to diagnose adverse ischemic event at 1 month.

ROC analysis showed that CBF presented the best performance among CTP and TCD variables assessed with an AUC standard error AUC(SE) of 0.81(0.08), p = 0.041 [Figure 2]; a CBF value of <24.5 presented 67% sensitivity and 100% specificity to diagnose adverse ischemic event at 1 month.

## Discussion

Our findings suggest that CTP is feasible in the everyday practice and it may provide useful information related to SAH-related vasospasm; and indeed, CTP and more particularly CBF could identify patients who presented late ischemic adverse events; this was more accurate than TCD at this point. In this respect, CTP is a promising diagnostic tool in the management of SAH.

Digital subtraction angiography is the gold standard in the diagnosis of CV and other cerebrovascular pathologies [18,19]. However, the invasiveness of the procedure and the exposure to radiation forbid its consecutive use and application to diagnosis [13]. A number of invasive methods, like microdialysis and intracerebral CBF measurement, have also been used for their value to identify CV in patients after SAH [13]. Those procedures avoid the risks of placement and long-term monitoring with intracerebral probes like infection and they are only precise if the measurement is performed in an area of the brain which will eventually be a?ected by decreased perfusion [13].

In addition, positron emission tomography, MR perfusion, and CTP, Xenon CT, and single-photon emission CT, all allow tomographic CBF assessment, while CTP is currently the most widely used and studied modality [20]. Various cutoff values that correlate with delayed CI have been reported, including an MTT exceeding 5.0–6.4 s, or regional CBF below 25–40 ml/100 g/min [21].

In this case series study, we observed two main different patient profiles based on patients TCD and CTP data. The first profile was associated with clinical outcome without ischemic lesions in CT at these areas, whereas the second was associated with poorer clinical outcome with infarct lesions in CT.

In the first one, patients had CTP which showed mild vasospasm according to CBF and MTT values, and TCD showed elevated PIs indicating severe vasospasm. This occurred in patients S2 and S5; S2 had in CTP only prolonged MTT from 7.3 ± 4 to 10 ± 2.8 and, in TCD, PIs were increased in the right and left ICA (1.2 and 1.3). In S5, CTP revealed a reduction in CBF with prolonged MTT, and TCD showed that PIs on the right & left were ACA = 0.6 & 1, MCA = 1.4 & 1.3, PCA = 0.8 & 1.6, mean PI = 1.1, which means mild vasospasm.

In the second profile, patients had CTP which showed severe vasospasm with clinical and CT findings suggesting ischemia (Table 1), but PI values in TCD were between 1.06 and 1.3, more compatible with mild vasospasm (i.e., patients 1, 3 and 4). In patient 1, CTP showed reduced CBF (in left frontal and parietal, right frontal areas), normal CBV values and prolonged MTT (7.4 ± 2 to 10.5 ± 3 s) and TCD with mean PI = 1.3. In patient 3, CTP revealed reduced CBV and CBF in right frontal, parietal areas and prolonged MTT mainly in the left side (7.4 ± 2 to 10.9 ± 3 s) and TCD with mean PI = 1.2. In patient 4, CTP showed reduction of CBF and prolonged MTT in the left from 7.4 ± 2 to 8.7 ± 2 s and TCD mean PI = 1.1 (Tables 2 & 3).

In our study we found that CTP indices presented better performance in ROC analysis compared with TCD values (Table 4). CBF reflects blood flow in the brain and any reduction of its values is closely related to the presence and severity of CV which is a significant predictor of clinical outcome as well. CTP indices seem to correlate fairly well with CI, but focal flow reductions can also occur as a consequence of brain retraction injury or perihematomal brain dysfunction [22]. TCD on the other hand provides significant information about the flow characteristics in an arterial segment and based on the spectral patterns in various adjacent intracranial arteries and may aid as a relatively simple screening method of CV; however, some authors were unable to find any correlation between TCD and angiographic results in patients with CV [23,24]. Also, TCD ultrasonography suffers from both technical and anatomical limitations and is operator dependent [25].

**Table 4.** **Statistical findings.**

**Statistical variables**	**Total CBF**	**Total CBV**	**Total MTT**	**Total PI**	**Total PSV**
Area	0.814	0.694	0.585	0.693	0.726

Standard error	0.080	0.117	0.122	0.083	0.122

p-value	0.041	0.206	0.577	0.360	0.195

CBF: Cerebral blood flow; CBV: Cerebral blood volume; MTT: Mean transit time; PI: Pulsatility Index; PSV: Peak systolic velocity.

CTP was used for evaluation of CI with sensitivities between 74.1 and 84% and specificities between 79 and 93.0% [26,27]. In our study all areas with reduced CBF were associated with late ischemic adverse events (AUC of 0.81[0.08], p = 0.041). This is in line with current literature, which reported that a decreased CBF may indicate irreversible ischemic lesion with a CBF threshold of <25 ml × 100 g^-1^ × min^-1^ whereas when CBF reduction is associated with CBV <2 ml × 100 g^-1^ or MTT is increased >145% penumbra, core infarct or can penumbra could be defined [28]. Our CBF threshold of 24.5 ml × 100 g^-1^ × min^-1^ was derived from traumatic and aneurysmal SAH and is similar to what was reported; in our study this threshold had 67% sensitivity and 100% specificity to diagnose adverse ischemic events at 1 month (Figure 2). A future larger study could evaluate whether CBF could be useful as a standalone diagnostic tool in this setting.

Previous studies [28] reported that a decreased CBF with a normal or increased CBV may also reflect hemodynamic hypoperfusion without a true ischemic lesion in response to the activation of the autoregulation.

An MTT alone may suggest mild–moderate vasospasm while MTT prolongation associated with rCBF and/or rCBV abnormalities may suggest severe vasospasm [28]. In our study, all patients who presented the above triad presented late ischemic adverse events, but we did not have a patient combining only MTT prolongation without being accompanied by some disturbance of the other CTP parameters and therefore we could not verify the usefulness of MTT as a standalone index. Instead in our case series study MTT prolongation associated with rCBF and/or rCBV abnormalities (patients 1, 3 & 4), related with clinical and CT evidence of ischemia.

The present study carries certain limitations. First, this is one center small study and the study population might be inhomogeneous; in this respect definitive conclusions for the role of CTP in the management of SAH cannot be drawn. Second, one might argue that a baseline CTP cannot be available or that CTP should have been performed earlier, within the first 24 h after bleeding, to avoid brain perfusion abnormalities related to other causes. Furthermore, angiographic studies were not available in this study and TCD may present limitations in detecting flow abnormalities in microcirculation, which could be responsible for CTP findings. We acknowledge that angiographic data could provide further insight in the diagnostic utility of CPT. However, such studies have been performed previously [10–12], and reports on CPT diagnostic performance have been underlined. The present study addressed the feasibility of CPT performance on the everyday clinical practice and aimed to evaluate its relationship with TCD; data on this field are very limited and our study may form the basis for a larger clinical study with a more homogeneous sample formed by aneurysmal or traumatic SAH in the future or to compare CPT to angiogram studies when performed and to assess if CBF predicts response to vasospasm treatment.

## Conclusion

In conclusion, these case series data provide evidence that CTP-derived CBF is a measurable index which may detect the degree of CI in a very early stage in patients suffering from SAH. Larger studies are needed in order to define better the role of CT perfusion in early diagnosis of CV.

## Future perspective

It is becoming clear that in patients suffering from SAH, TCD combined with CTP helps with the detection of the degree of CI at a very early stage. In future, larger clinical studies are needed with more homogeneous samples formed by aneurysmal or traumatic SAH, along with comparison of CPT to angiogram studies and to assessment of whether CBF predicts response to vasospasm treatment. To this end, CTP and other examinations may prove to be highly effective in identifying which patients with SAH should receive additional therapy in order to avoid CV.

**Summary points**

- We could not verify the usefulness of mean transit time as a standalone index for mild–moderate vasospasm.
- Computed tomography perfusion-derived cerebral blood flow is a measurable index that may detect the degree of cerebral ischemia at a very early stage in patients suffering with subarachnoid hemorrhage.
